# Retinoic Acid Protects and Rescues the Development of Zebrafish Embryonic Retinal Photoreceptor Cells from Exposure to Paclobutrazol

**DOI:** 10.3390/ijms18010130

**Published:** 2017-01-11

**Authors:** Wen-Der Wang, Hwei-Jan Hsu, Yi-Fang Li, Chang-Yi Wu

**Affiliations:** 1 Department of Bio-Agricultural Sciences, National Chiayi University, Chiayi City 60004, Taiwan; s1002440@mail.ncyu.edu.tw; 2 Institute of Cellular and Organismic Biology, Academia Sinica, Taipei City 11529, Taiwan; cohsu@gate.sinica.edu.tw; 3 Department of Biological Sciences, National Sun Yat-Sen University, Kaohsiung City 80424, Taiwan; cywu@mail.nsysu.edu.tw

**Keywords:** paclobutrazol, retina photoreceptor cells, zebrafish, retinoic acid

## Abstract

Paclobutrazol (PBZ) is a widely used fungicide that shows toxicity to aquatic embryos, probably through rain-wash. Here, we specifically focus on its toxic effect on eye development in zebrafish, as well as the role of retinoic acid (RA), a metabolite of vitamin A that controls proliferation and differentiation of retinal photoreceptor cells, in this toxicity. Embryos were exposed to PBZ with or without RA from 2 to 72 h post-fertilization (hpf), and PBZ-treated embryos (2–72 hpf) were exposed to RA for additional hours until 120 hpf. Eye size and histology were examined. Expression levels of *gnat1* (rod photoreceptor marker), *gnat2* (cone photoreceptor marker), *aldehyde dehydrogenases* (encoding key enzymes for RA synthesis), and phospho-histone H3 (an M-phase marker) in the eyes of control and treated embryos were examined. PBZ exposure dramatically reduces photoreceptor proliferation, thus resulting in a thinning of the photoreceptor cell layer and leading to a small eye. Co-treatment of PBZ with RA, or post-treatment of PBZ-treated embryos with RA, partially rescues photoreceptor cells, revealed by expression levels of marker proteins and by retinal cell proliferation. PBZ has strong embryonic toxicity to retinal photoreceptors, probably via suppressing the production of RA, with effects including impaired retinal cell division.

## 1. Introduction

Pesticides are applied across entire agricultural fields; they affect not only their target crops but also other organisms around the field. Triazole pesticides, which are more environmentally friendly, are commonly substituted for organochlorine pesticides [1]. However, triazole pesticides have low biodegradability, and high chemical stability and water solubility, resulting in their circulation in the environment [2]. Several reports have revealed that residual triazole pesticides can accumulate in aquatic organisms via bioaccumulation and biotransformation [1,3].

Paclobutrazol, (2*RS*,3*RS*)-1-(4-chlorophenyl)-4,4-dimethyl-2-(1*H*-1,2,4–triazol-1-yl) pentan-3-ol (henceforth referred to as PBZ), is a triazole pesticide containing a single aromatic ring and is frequently used as a plant growth retardant because it inhibits the synthesis of the plant hormone gibberellin, thereby regulating plant growth and acting as an antifungal [4]. To date, PBZ has been in use for 30 years, since 1986. The wide and long-term overuse of PBZ in the wild has led to ecological contamination, including terrestrial and aquatic environments. Surprisingly, PBZ residue in the surface water of the Jiulong River Estuary and the Western Xiamen Sea, China, was approximately 119.6 ppb [5]. Unfortunately, the toxicology of PBZ on aquatic organisms remains poorly understood. Only a few scientific studies have indicated that PBZ exposure leads to the induction of antioxidant enzyme activity in adult zebrafish [6] and to hepatic steatosis in *Sebastiscus marmoratus* [7,8]. Our previous studies have also reported that PBZ disturbs the morphological development of the head, eyes, and heart in zebrafish embryos [9], and that PBZ disrupts the development of the zebrafish digestive system, including the liver, intestine, and pancreas, by activating aryl hydrocarbon receptor 2 signaling [10]. Here, we investigated the impairment of PBZ on the development of retinal cells.

The vertebrate retina contains two types of specialized neurons, rod and cone photoreceptors, which are optimized for low- and high-intensity light, respectively [11]. Retinal rod and cone photoreceptors contribute to the light intensity and color information used by the visual system to form a representation of the visual world [12]. The zebrafish (*Danio rerio*) is a powerful model system for in vivo toxicity testing, offering efficiency and reliability for identifying the biological effects and mechanisms of toxicity [13]. During embryogenesis, morphogenesis of the eye and lens, retinal histology, and the transcription factors and regulatory mechanisms involved in eye development are conserved between zebrafish and other vertebrates [14,15,16,17,18]. After subsequent morphogenetic movements and inductive interactions, the bilateral paddle-shaped masses of cells are formed from the forebrain [19], and the optic cup is formed after further invagination of the central portions of the eye-mass [20]. The inner layers of cells continue to proliferate and differentiate to produce the retinal rod and cone photoreceptors, and the outer layer gives rise to the retinal pigment epithelium [21]. The developing retinal rod and cone cells can be identified based on gene expression and morphology [22,23].

Retinoic acid (RA), a diffusible lipophilic molecule that acts as a ligand for nuclear RA receptors, is a metabolite of vitamin A (retinol) that mediates the functions of vitamin A and is required for growth and development [24,25]. During eye development, RA plays critical roles in the regulation of optic primordium formation, cell proliferation, and cell differentiation [26,27,28]. In addition, application of excessive RA promotes cell proliferation in the eye and can even result in a duplication of the retina [29]. Furthermore, ectopic addition of RA also alters the development of rod and cone cells in vivo [30,31]. In embryos, aldehyde dehydrogenases (Aldhs), especially Aldh1a2 and Aldh1a3, are the key enzymes that catalyze RA synthesis from retinaldehyde, a middle metabolic product of vitamin A [32]. In rat and zebrafish embryos, RA is highly synthesized in developing retina [33]. In vertebrate embryos, RA distribution is spatially and temporally regulated by the localized expression of Aldhs, which form the origins of RA diffusion gradients in the surrounding tissues. Many lines of evidence indicate that disruption of vitamin A (or RA)-mediated signaling leads to defective development or degeneration of retinal photoreceptor cells [30,34,35,36].

In this study, we show that PBZ exposure causes small eyes accompanied by the reduction of the photoreceptor cell layer, as well as the reduced expression level and territory of rod and cone cell markers, in a dose-dependent manner. In addition, expression levels of Aldh1 and Aldh3, enzymes for RA synthesis, are severely decreased in PBZ-treated eyes, suggesting a deficiency of RA. Strikingly, supplementation of RA in embryos treated with PBZ, or in embryos after PBZ treatment, partially rescues eye size and expressions of rod and cone markers. Furthermore, PBZ does not induce cell death while disrupting cell division, which can be rescued by RA addition. Our results clearly document the toxic effect of PBZ on the synthesis of RA, which controls the proliferation of photoreceptors, and such effects may also exist in other organisms that are exposed to PBZ.

## 2. Results

### 2.1. Paclobutrazol Exposure Dose-Dependently Reduces Eye Size in Zebrafish Embryos

**Figure 1:**
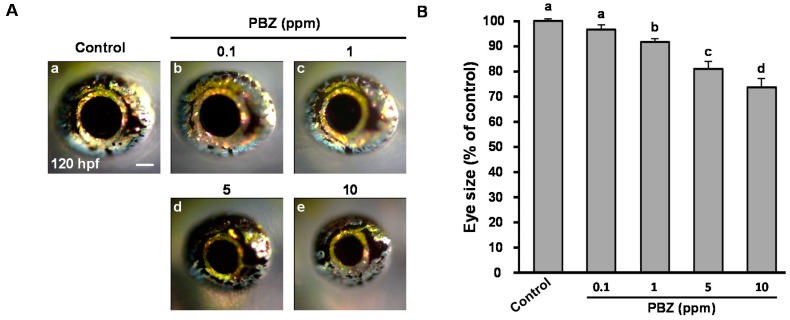
Paclobutrazol (PBZ) exposure reduces eye size in zebrafish embryos. (**A**) Representative eye photomicrographs (20× magnification) from 120 hours post-fertilization (hpf) embryos treated with (**a**) 0.1% DMSO (control) or (**b**) 0.1 ppm; (**c**) 1 ppm; (**d**) 5 ppm; or (**e**) 10 ppm of PBZ. Scale bar: 10 μm; (**B**) Eye areas from 15 embryos treated with 0.1% DMSO or with 0.1, 1, 5, or 10 ppm of PBZ were measured using ImageJ software, and all values were normalized to the mean of the control group. Bars sharing a letter are not significantly different from one another at *p* < 0.05, as assessed by one-way ANOVA, followed by Fisher’s least significant difference test. Error bars indicate standard error.

To study the toxic effects of PBZ on the eye development of zebrafish, we exposed embryos to 0.1, 1, 5, and 10 ppm of PBZ, at concentrations lower than its medial concentration 50 (LC_50_) on zebrafish embryos, 20 ppm, which we identified previously [10]. Notably, under these conditions, survival rates and the body size were comparable with 0.1% dimethyl sulfoxide (DMSO)-treated control embryos [10]. Intriguingly, at 120 hpf, the size of eyes in PBZ-exposed embryos was significantly reduced (Figure 1A), and in a dose-dependent manner (Figure 1B). Compared to control embryos, the eye size was not obviously reduced in embryos treated with PBZ at 0.1 ppm, but was 8.11% ± 1.08%, 19.54% ± 1.67% and 38.03% ± 4.58% reduced in embryos treated with 1, 5, or 10 ppm PBZ (*p* < 0.001), respectively. Given that 10 ppm PBZ-treated embryos were also accompanied by the sever cardiac and yolk sac edema [9,10], to specifically address the effect of PBZ on eye development, we did not use 10 ppm-treated embryos further in this study.

### 2.2. Toxic Effects of PBZ on the Development of Retinal Photoreceptor Cells

**Figure 2:**
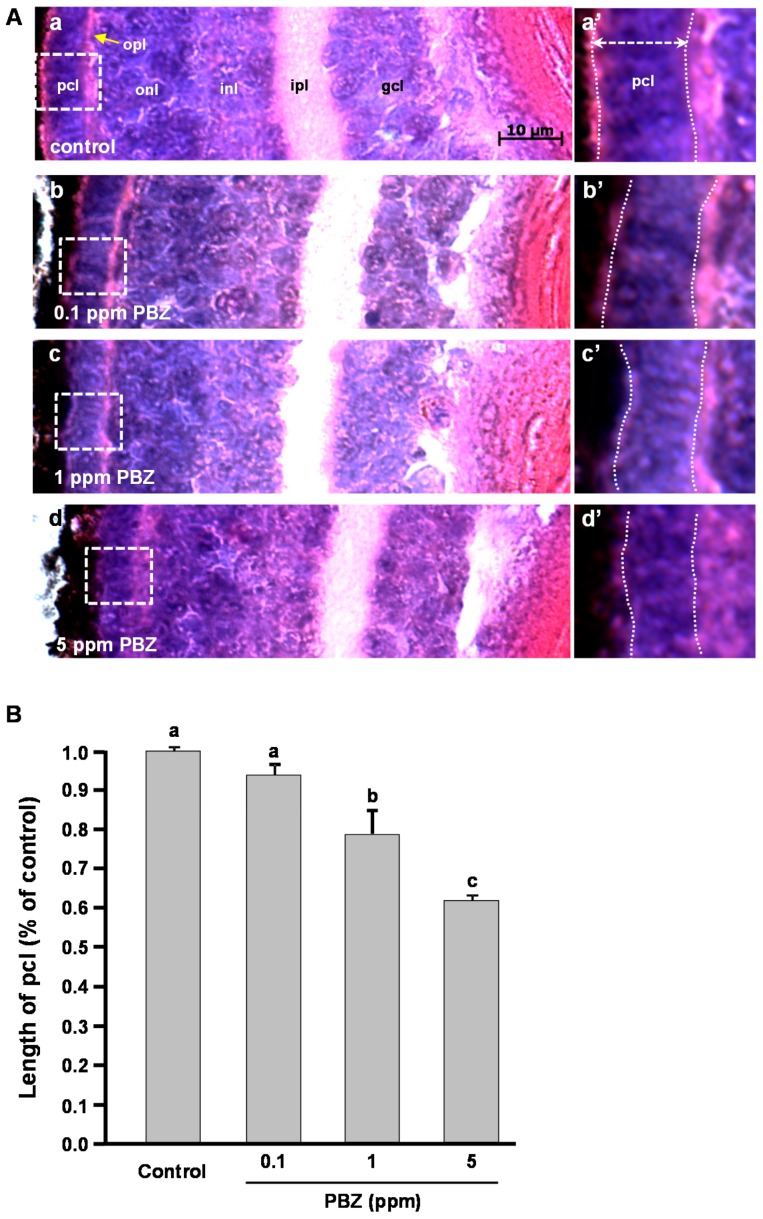
Paclobutrazol exposure significantly reduces the thickness of the photoreceptor layer in zebrafish embryos. (**A**) Hematoxylin and eosin (H&E) staining of eye sections from zebrafish treated with (**a**) 0.1% DMSO (control) or with (**b**) 0.1 ppm; (**c**) 1 ppm; or (**d**) 5 ppm. High-magnification images for the photoreceptor layer of the eyes are shown in **a’**–**d’**. Reference lines indicate the photoreceptor layer. gcl, ganglion cell layer; inl, inner nuclear layer; ipl, inner plexiform layer; onl, outer nuclear layer; opl, outer plexiform layer; pcl, photoreceptor cell layer; (**B**) Thicknesses of the photoreceptor cell layer were measured from control embryos and embryos treated with PBZ at 0.1, 1, or 5 ppm, with 20 embryos used per condition. Each group contains at least 10 embryos; Scale bar: 10 μm. All values were normalized to the mean of the normal group. Bars sharing a letter are not significantly different from one another as assessed by one-way ANOVA, followed by Fisher’s least significant difference test (*p* < 0.05). Error bars indicate standard error.

To dissect the toxic effects of PBZ on eye development, PBZ-treated embryos were analyzed by histopathology imagery (Figure 2A). In the control, (Figure 2Aa), the eye consisted of a well-organized ganglion cell layer (gcl), inner plexiform layer (ipl), inner nuclear layer (inl), outer nuclear layer (onl), outer plexiform layer (opl), and a photoreceptor cell layer (pcl). However, this cell arrangement was affected in embryos treated with 0.1, 1, or 5 ppm of PBZ (Figure 2Ab–d). However, the individual layer of the eye was formed in PBZ-treated embryos, and only the photoreceptor cell layer was obviously thinner in embryos treated with 1 or 5 ppm PBZ (Figure 2Ac’–d’), as compared to the control or to the 0.1 ppm PBZ-treated embryos. By measuring the thickness of the photoreceptor cell layer, we also found that 1 (53.85 ± 4.32 μm, *n* = 10, *p* < 0.05) or 5 ppm PBZ (43.92 ± 5.61 μm, *n* = 10, *p* < 0.001) significantly decreased the thickness of the photoreceptor cell layer to 20% and 37% of that in the control eyes (69.23 ± 3.14 μm, *n* = 10), respectively, showing a dose-dependent effect (Figure 2B).

By the normalization with the values of wild-type eyes, 1 and 5 ppm PBZ-treated eyes showed 15% and 20% reduction in volume, which was exhibited significantly. Similarly, the thickness of the photoreceptor cell layer in 1 and 5 ppm PBZ-treated eyes also showed 20% and 38% reduction, as compared to the control (*p* < 0.001). Although the highest reduction is only 38%, it reaches statistically significant differences.

**Figure 3:**
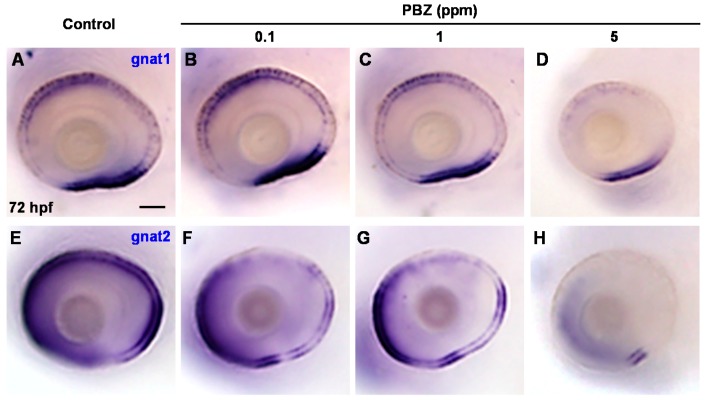
Paclobutrazol interferes the development of embryonic photoreceptor cells in zebrafish. Eyes of 72 hpf embryos treated with (**A**,**E**) 0.1% DMSO (control) or with (**B**,**F**) 0.1 ppm, (**C**,**G**) 1 ppm, or (**D**,**H**) 5 ppm of PBZ and labeled with riboprobes for gnat1 (rod cell marker) (**A**–**D**) or gnat2 (cone cell marker) (**E**–**H**) by in situ hybridization. Each group contains at least 20 embryos; Scale bar: 50 μm.

The photoreceptor cell layer is mainly composed of rod and cone cells, which function in light and color vision, respectively [37]. To determine whether PBZ affects the development of retinal rod and cone cells, we examined the expression of gnat1 (a rod cell marker) and gnat2 (a cone cell marker) in 72 hpf embryos by in situ hybridization. Our results showed that the expression level and territory of gnat1 in embryos treated with 0.1 ppm PBZ was similar to that in control embryos (Figure 3A,B). In contrast, embryos treated with 1 ppm PBZ exhibited slightly reduced expression intensity and domain of gnat1; however, 5 ppm-treated embryos showed strong disruption of gnat1 expression level and territory (Figure 3C,D). On the other hand, compared to control embryos, expression of gnat2 was slightly reduced in embryos treated with 0.1 or 1 ppm PBZ and was severely reduced in embryos treated with 5 ppm PBZ (Figure 3E–H). This result suggests that PBZ, at least at a concentration of 5 ppm, affects photoreceptor cell formation via the effect on their division, survival, or differentiation.

### 2.3. Reduction of Retinoic Acid Synthesis Is Involved in the Defect in Photoreceptor Cells of Paclobutrazol-Treated Embryos

**Figure 4:**
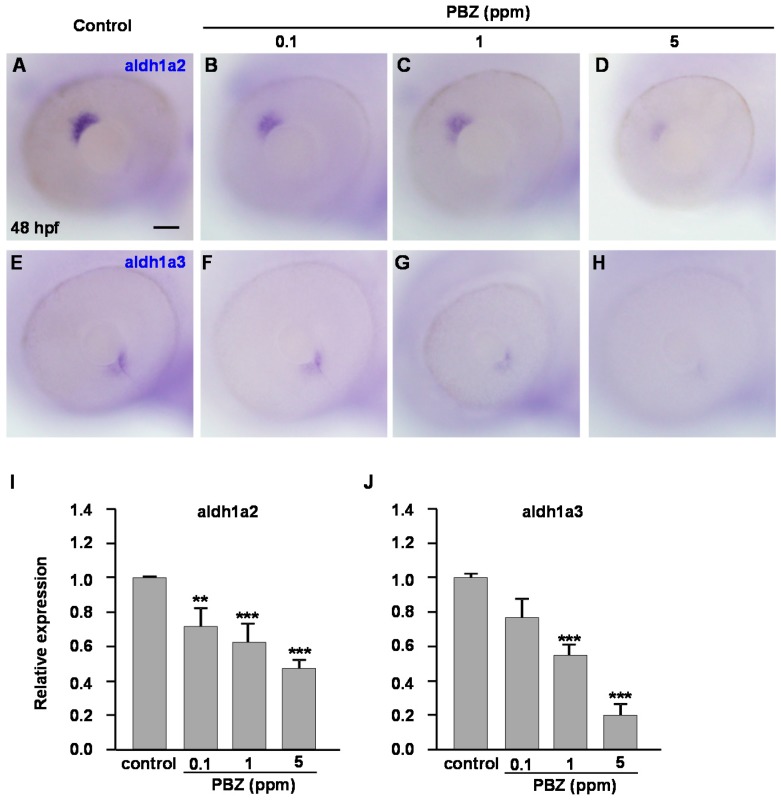
Expression of aldehyde dehydrogenases (aldh1a2 and aldh1a3), encoding key enzymes for retinoic acid (RA) synthesis, is decreased in PBZ-treated embryos. Whole-mount in situ hybridization was used to examine the expression of aldh1a2 (**A**–**D**) and aldh1a3 (**E**–**H**) in the eyes of 48 hpf zebrafish embryos treated with (**A**,**E**) 0.1% DMSO (control) or with (**B**,**F**) 0.1 ppm, (**C**,**G**) 1 ppm, or (**D**,**H**) 5 ppm of PBZ. (**I**,**J**) Quantitative PCR analysis of the aldh1a2 (**I**) and aldh1a3 (**J**) mRNA levels in embryos treated with 0.1% DMSO or 0.1, 1, or 5 ppm PBZ at 48 hpf. Each group contains at least 20 embryos; Scale bar: 50 μm. Error bars represent standard deviation. Data were analyzed using Student’s *t*-test; ** *p* < 0.01 and *** *p* < 0.001.

Retinoic acid is a biologically active metabolite of vitamin A and plays multiple roles in embryonic eye development [26]. In *Xenopus* embryos, exogenous addition of RA causes the duplication of the retina, and pharmacological reduction of RA signaling results in severe defects in retinal patterning [31]. In zebrafish, RA signaling is known to control differentiation and maturation of both rod and cone photoreceptors [29,33]. Therefore, we first examined the expression of aldh, encoding the enzyme for RA biosynthesis, by in situ hybridization staining. Compared to the control, expression of aldh1a2 was reduced in embryos treated with 0.1, 1, or 5 ppm PBZ (Figure 4A–D), while expression of aldh1a3 was not obviously altered in embryos treated with 0.1 ppm PBZ and was significantly reduced in embryos treated with 1 or 5 ppm PBZ (Figure 4E–H). Our quantitative reverse transcription polymerase chain reaction (qRT-PCR) data also confirm these results (Figure 4I,J). The reduction of aldh1a2 and aldh1a3 expression in PBZ-treated embryos suggests that PBZ exposure may lead to a reduction of RA synthesis, which in turn leads to developmental defects in retinal photoreceptor cells.

**Figure 5:**
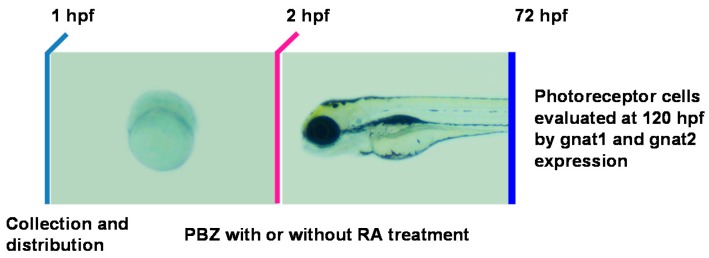
Schematic diagram showing the timeline of PBZ exposure, RA treatment, and collection of embryos for analysis. Embryos were independently exposed to PBZ (0, 1, or 5 ppm) with or without RA (1 or 5 nM) from 2 hpf until 72 hpf. At 72 hpf, embryos were collected for analysis of retinal photoreceptor cells via gnat1 and gnat2 in situ hybridization.

To address the role of RA in the PBZ-induced photoreceptor cell defects, zebrafish embryos were incubated in PBZ (0, 0.1, 1, or 5 ppm) with or without RA (1 or 5 nM) at 2 hpf and were harvested at 72 hpf for in situ hybridization with gnat1 or gnat2 antisense riboprobes to monitor the development of rod and cone photoreceptor cells, respectively (Figure 5).

**Figure 6:**
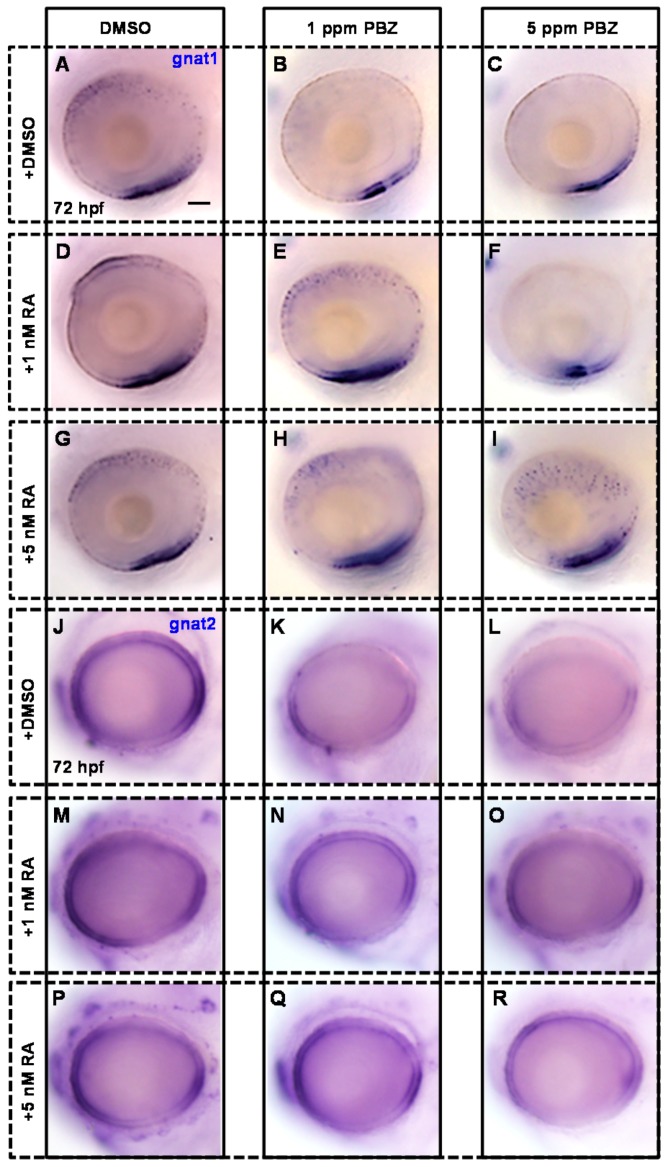
Retinoic acid increases embryos’ tolerance to the toxic effects of PBZ on retinal photoreceptor development. Fertilized embryos were incubated with (**A**,**J**) 0.1% DMSO (control); (**B**,**K**) 1 ppm PBZ; (**C**,**L**) 5 ppm PBZ; (**D**,**M**) 1 nM RA; (**E**,**N**) 1 ppm PBZ + 1 nM RA; (**F**,**O**) 5 ppm PBZ + 1 nM RA; (**G**,**P**) 5 nM RA; (**H**,**Q**) 1 ppm PBZ + 5 nM RA; or (**I**,**R**) 5 ppm PBZ + 5 nM RA from 2 to 72 hpf. The development of retinal photoreceptor cells was analyzed by in situ hybridization with digoxigenin-labeled gnat1 and gnat2 cRNA probes at 72 hpf. Each group contains at least 20 embryos; Scale bar: 50 μm.

Consistently, compared to the control (Figure 6A), the expression level and domain of gnat1 were obviously reduced in embryos treated with 1 or 5 ppm PBZ (Figure 6B,C), but were not changed in embryos exposed to 1 or 5 nM RA alone (Figure 6D,G). Strikingly, the reduced expression level and domain of gnat1 in 1 ppm PBZ-treated embryos were partially rescued by adding RA at 1 or 5 nM (Figure 6E,H). Furthermore, the severe reduction of gnat1 expression in embryos treated with 5 ppm PBZ was not rescued by adding 1 nM RA (Figure 6F), but was greatly enhanced by the addition of 5 nM RA (Figure 6I). Notably, 5 nM RA also induced ectopic expression of gnat1 in embryos treated with 5 ppm PBZ (Figure 6I). These results suggest that supplementation of RA partially rescues the PBZ-induced impairment of rod cell development. Similarly, compared to the control (Figure 6J), expression of gnat2 was mildly and severely reduced in embryos treated with 1 and 5 ppm PBZ, respectively (Figure 6K,L), while gnat2 expression was not changed in embryos exposed to 1 or 5 nM RA alone (Figure 6M,P). Interestingly, gnat2 expression was slightly restored by 1 nM RA, and was better rescued by 5 nM RA in embryos treated with either 1 or 5 ppm PBZ (Figure 6N,O,Q,R), suggesting that RA addition also partially rescued the PBZ-induced defect of cone cells. Taken together, these results indicate that PBZ-induced retinal damage is mediated by interference with RA signaling.

**Figure 7:**
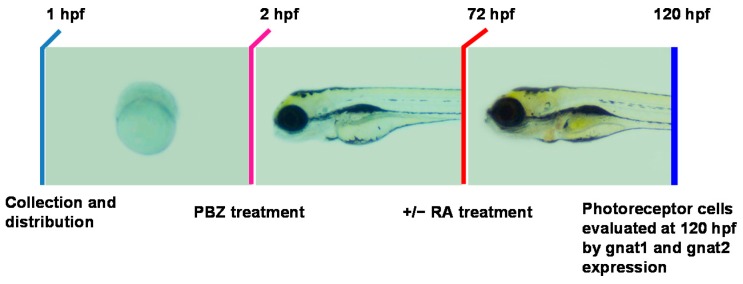
Schematic diagram showing the timeline of PBZ exposure, the addition of RA, and the collection of embryos for analysis. Embryos were exposed separately to different PBZ concentrations (0, 1, or 5 ppm) from 2 to 72 hpf. After the removal of PBZ, 1 or 5 nM RA was added to the embryos’ water for an additional 48 h. At 120 hpf, embryos were collected and fixed for further examination and evaluation of retinal photoreceptor development.

Next, we investigated whether RA could repair PBZ-induced photoreceptor cell injury. To address this, embryos were exposed to PBZ from 2 to 72 hpf. After PBZ treatment, embryos were incubated with RA for another 48 h and were then subjected to in situ hybridization to identify photoreceptor cells as described above (Figure 7).

**Figure 8:**
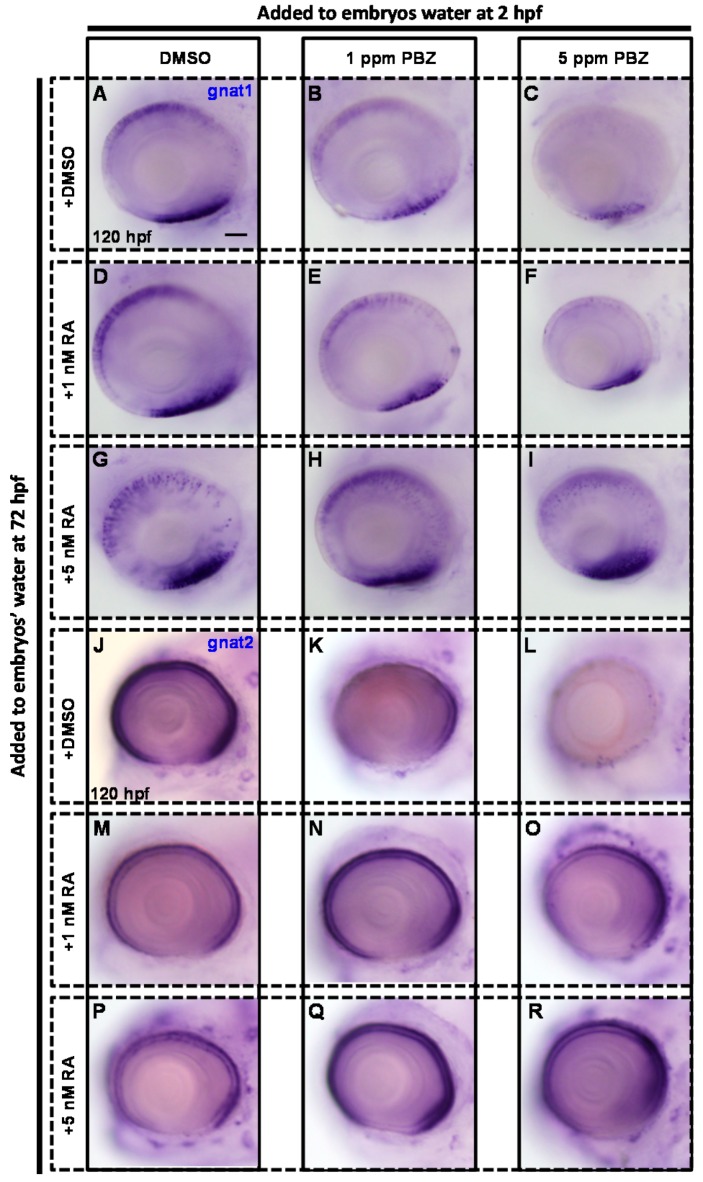
Paclobutrazol-damaged retinal photoreceptor cells are restored by treatment with RA. Fertilized embryos were incubated with (**A**,**J**) 0.1% DMSO (control); (**B**,**K**) 1 ppm PBZ; (**C**,**L**) 5 ppm PBZ; (**D**,**M**) 1 nM RA; (**E**,**N**) 1 ppm PBZ + 1 nM RA; (**F**,**O**) 5 ppm PBZ + 1 nM RA; (**G**,**P**) 5 nM RA; (**H**,**Q**) 1 ppm PBZ + 5 nM RA; or (**I**,**R**) 5 ppm PBZ + 5 nM RA. The development of retinal photoreceptor cells was analyzed by in situ hybridization with digoxigenin-labeled gnat1 (**A**–**I**) and gnat2 (**J**–**R**) cRNA probes at 120 hpf. Note that RA was added to the embryo for 48 h, beginning at 72 hpf. Scale bar: 50 μm.

Similar to the results showing injured retinal rod and cone cells in PBZ-treated embryos at 72 hpf (see Figure 6A–C), PBZ-induced retinal injury was also observed in the embryos treated with PBZ for 120 h (Figure 8A–C). Compared to the control, embryos exposed to 1 nM RA for 120 h exhibited normal expression of gnat1 (Figure 8D), while 5 nM of RA induced ectopic expression of gnat1 (Figure 8G). Additional treatment with 1 nM or 5 nM RA clearly produced a partial rescue of gnat1 expression in PBZ-treated embryos (Figure 8E,F,H,I), although gnat1 was ectopically expressed in PBZ-treated embryos with additional addition of 5 nM RA (Figure 8H,I). Our results revealed that the addition of 1 nM retinoic acid was sufficient to rescue the PBZ-induced impairment of rod cell development. Similar experiments were performed to analyze the role of RA in cone cell development after PBZ treatment (Figure 8J–R). The development of cone cells was obviously affected in embryos exposed to 1 or 5 ppm PBZ from 2 hpf to 120 hpf compared to the controls (Figure 8J,K,L). Expression of gnat2 was significantly reduced in embryos treated with 1 or 5 nM RA alone (Figure 8M,P). Interestingly, gnat2 expression in embryos treated with 1 or 5 ppm PBZ was rescued by additional treatment with 1 or 5 nM RA (Figure 8N,O,Q,R). These results indicate that RA treatment also rescued PBZ-impaired cone cell development.

**Figure 9:**
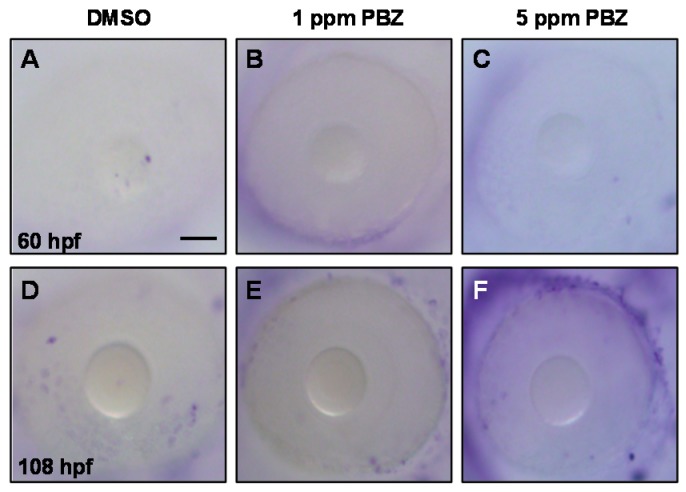
Paclobutrazol treatment does not induce cell death in the eye. Fertilized embryos were incubated with (**A**) 0.1% DMSO (control); (**B**) 1 ppm PBZ; (**C**) 5 ppm PBZ until 60 hpf (**A**–**C**) and 108 hpf (**D**–**E**), and terminal deoxynucleotidyl transferase dUTP nick-end labeling (TUNEL) assay was performed for apoptotic cell analysis. Arrowheads indicate dead cells. Scale bar: 50 μm.

**Figure 10:**
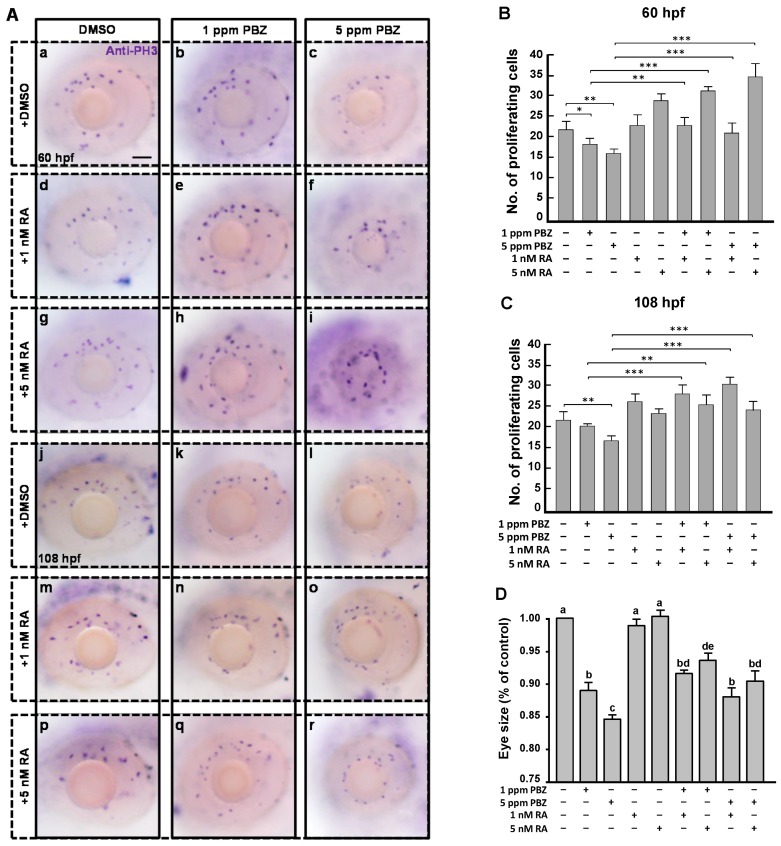
Retinoic acid treatment rescues reduced mitotic cells and eye size of PBZ-treated embryos. Embryos exposed to PBZ concentrations (0, 1, or 5 ppm) with or without 1 nM or 5 nM RA was performed at 2 hpf in embryos’ water. (**A**) Immunostaining was performed to analyze the retinal cell proliferation using an anti-phospho-histone H3 (PH3) antibody at the embryonic stage of 60 (**a**–**i**) and 108 hpf (**j**–**r**). Scale bar: 50 μm. The proliferating cell numbers were counted and recorded at (**B**) 60 and (**C**) 108 hpf. * *p* < 0.05, ** *p* < 0.01, and *** *p* < 0.001. (**D**) Eye areas from 15 embryos were measured using ImageJ software, and all values were normalized to the mean of the control group. Bars sharing a letter are not significantly different from one another at *p* < 0.05, as assessed by one-way ANOVA, followed by Fisher’s least significant difference test. Error bars indicate standard error.

To know if the reduced retinal photoreceptor cells caused by PBZ exposure are due to induced cell death, we performed a terminal deoxynucleotidyl transferase dUTP nick-end labeling (TUNEL; Roche Applied Science, Indianapolis, IN, USA) assay. Our results showed that PBZ did not induce cell apoptosis in the eye (Figure 9). We further examined if PBZ disrupts cell proliferation in the eye by examining the numbers of cells that were at the mitosis phase using an anti-phospho-histone H3 (PH3) antibody, which recognizes condensed chromosomes that occur during mitosis (Figure 10A). Our results clearly showed that PBZ reduced retinal cell division in embryos at 60 and 108 hpf (Figure 10A–C), indicated by the lower number of PH3-positive cells present in the eyes as compared to the control. Treatment with 1 and 5 nM alone did not promote cell division in embryos at 60 and 108 hpf (Figure 10B,C). Strikingly, exogenous supplementation with RA significantly recovered the division defects caused by PBZ treatment (Figure 10A–C). In addition, the reduction of eye size in PBZ-treated embryos was also partially rescued by RA treatment (Figure 10D). Since RA treatment could rescue expression of rod and cone cell markers (see Figure 6) via its effects on cell division, this suggests that the width of photoreceptor layer is also partially rescued. This result also suggests that PBZ may not directly affect rod and cone cell differentiation; instead, PBZ reduces the expression of aldh, which leads to a low level of RA and results in the reduction of retinal cell proliferation.

## 3. Discussion

The widespread use of pesticides, including PBZ, results in environmental pollution and is detrimental to wild organisms and to human public health [38,39,40,41,42,43], while few studies address their effects to aquatic animals. In this study, we have demonstrated that PBZ harms retinal photoreceptor cell division via the RA-mediated signaling pathway.

### 3.1. Paclobutrazol Has Wild Toxic Effects on Fish Embryo Development

In our previous studies, we have shown that PBZ strongly reduces zebrafish embryo hatching and survival rate [9,10]. In addition, PBZ affects the heart looping process, and thus causes heart failure and pericardial edema. Paclobutrazol also disturbs the development of digestive organs, including the liver, intestine, and pancreas. Furthermore, PBZ treatment causes deformed craniofacial cartilages, probably due to its effect on neural crest migration. Here, we have also shown that PBZ affects retinal photoreceptor cell development. Although we do not know how PBZ affects heart and craniofacial cartilage development, PBZ affects digestive organs and retinal photoreceptor cells via aryl hydrocarbon receptor 2 and RA mediated signaling, respectively. These results suggest that PBZ has wild toxic effects on different cell types of aquatic embryos, and probably via different targets to trigger different signaling pathways.

### 3.2. Paclobutrazol Affects Retinoic Acid Synthesis

During embryogenesis, retinoic acid plays an important role in the regulation of various aspects of embryonic morphogenesis [44,45,46,47,48], including eye development and retinal photoreceptor cell maintenance and proliferation [26,28,31,49,50,51]. During embryogenesis, retinoic acid synthesis originates with maternal retinol (or egg-stored retinoids) through the retinol dehydrogenase and aldehyde dehydrogenase enzymatic pathways [32,52]. Reducing the enzymatic activity within the pathway by using pharmacological inhibitors leads to decreased production of retinoic acid and results in the developmental hypoplasia of the retina [33], and the absence of RA signaling in *aldh* mutants results in severe ocular defects [53]. In our study, the endogenous expression of *aldh1a2* and *aldh1a3* was markedly reduced after PBZ exposure, and the PBZ-induced defects in retinal rod and cone cells can be partially ameliorated by the combined treatment of embryos with retinoic acid and PBZ or can be rescued by the addition of retinoic acid to embryos previously treated with PBZ. These results indicate that PBZ affects the production of RA, via the control of *aldh* expression at a transcription level. However, it remains unclear how PBZ affects *aldh* expression.

Our results provide valuable information for a more comprehensive understanding of the toxic effects of PBZ during embryogenesis. Further studies are needed to better understand the toxic effects and the toxic mechanism of PBZ in order to have a broad overview of the impacts of this pesticide on embryonic development.

## 4. Materials and Methods

### 4.1. Ethics Statement

The Institutional Animal Care and Use Committee (IACUC) at National Chiayi University approved (No. 104043) this study plan for the proper use of zebrafish. All experiments were performed according to the guidelines of the IACUC.

### 4.2. Fish Maintenance, Embryo Collection, and Treatments

Zebrafish (AB) were raised and kept under standard laboratory conditions at 28.5 °C [54]. Wild-type embryos were collected and washed by the 8-cell stage; embryos were subsequently cultured in egg water (0.0735% sea salt in deionized distilled water) containing PBZ at various concentrations (0.01 ppm (0.34 μM), 1 ppm (3.4 μM), and 5 ppm (17 μM)) or 0.1% (*v*/*v*) DMSO (control) at 28.5 °C. Culture media were not replaced during experiments until the embryos were fixed in 4% paraformaldehyde (PFA) dissolved in phosphate buffered saline (PBS) for further analysis. In vitamin A rescue experiments, at 72 hpf, the culture media were replaced with fresh media containing 0.1% DMSO or PBZ (at 0.1, 1, or 5 ppm), and/or 1 or 5 nM retinoic acid for two more days, at which point the embryos were fixed in 4% PFA. For in situ hybridization and immunostaining experiments, embryos were treated with 1-phenyl-2-thiourea (final concentration of 0.2 mM; Sigma–Aldrich, St. Louis, MO, USA) at 20–22 hpf to suppress pigmentation and were staged as described previously [55]. The fixed embryos were dehydrated and kept in 100% methanol at −20 °C for future experimental analysis.

### 4.3. Chemicals

Paclobutrazol, (2*RS*,3*RS*)-1-(4-chlorophenyl)-4,4-dimethyl-2-(1*H*-1,2,4-triazol-1-yl) pentan-3-ol, was purchased from Sigma-Aldrich. Stock solution of PBZ at a concentration of 300,000 ppm was dissolved in DMSO and stored at −20 °C for experimental use.

### 4.4. Whole-Mount In Situ Hybridization, Immunochemistry, and Cell Death Assay

For in situ hybridization and immunochemical staining analyses, dehydrated embryos were gently rehydrated to PBT (PBS containing 0.1% Tween 20). Whole-mount in situ hybridization was performed as described previously [56]. Gene-specific antisense probes labeled with digoxigenin-UTP (Roche Applied Science) were synthesized using cDNA templates encoding gnat1 [56], gnat2 [56], aldh1a2 [57], or aldh1a3 [58] by in vitro transcription reactions. The gene expression signals were developed using nitro blue tetrazolium/5-bromo-4-chloro-3-indolyl phosphate (NBT/BCIP). For immunostaining, embryos were incubated with anti-phospho-H3 (1:500; GeneTex, San Antonio, TX, USA) antibodies overnight at 4 °C. After washing, embryos were exposed to horseradish peroxidase (HRP)-conjugated secondary antibodies (1:5000) for another hour at room temperature and washed again. Signals were developed using 3,3′-diaminobenzidine (DAB) as a substrate (Vector Laboratories, Burlingame, CA, USA). TUNEL assay was performed as described elsewhere [9].

### 4.5. Histological Study

Embryos were fixed with Bouin’s solution and embedded in 1% agarose. Agarose blocks were then dehydrated with 70%, 95%, and 100% ethanol, 30 min for each solution, equilibrated in Histoclear II (National Diagnostics, Atlanta, GA, USA) for 1 h, and then in paraffin three times for 30 min at 56 °C. The block was then embedded in paraffin in the desired orientation on a holder and allowed to cool to room temperature, and was stored in 4 °C. Samples were sectioned at 6-mm thickness and stained with hematoxylin and eosin for morphological observation.

### 4.6. Quantitative Reverse Transcription Polymerase Chain Reaction Analysis

Total RNA was extracted using Trizol reagent (Invitrogen, Philadelphia, PA, USA). Synthesis of cDNA was performed using 3 μg of total RNA. Quantitative PCR reactions were performed with iQ™ SYBR Green Supermix (BioRad, Hercules, CA, USA). Quantitative RT-PCR analysis was performed using the Rotor-Gene Q System (QIAGEN, Valencia, CA, USA), and gene expression levels for each individual sample were normalized to actin. Results were analyzed using a previously described formula [59]. The following forward/reverse primers were used: actin: 5’-GACTCAGGATGCGGAAACTG-3’, 5’-GAAGTCCTGCAAGATCTTCAC-3’; aldh1a2: 5’-TTGTCCTGAAACCTGCTGAG-3’, 5’-GCTCTTTCCTGCTGCTTCTT-3’; and aldh1a3: 5’-AGGGCAAACAGCTCTCAGTT-3’, 5’-GGCTTTGACCTCGGTGTATT-3’.

### 4.7. Image and Statistical Analysis

Eye areas and the width of photoreceptor layers were analyzed by Image J [60]. Data were subjected to the ANOVA and are presented as the mean ± standard deviation. For the eye area and cell proliferation, pairwise significant difference between the treatment groups were evaluated using the one-way ANOVA followed by the Fisher’s least significant difference test (*p* < 0.05). All analyses were performed using SSPS 21.0 (IBM, Armonk, NY, USA).

## 5. Conclusions

In this study, we have shown that PBZ causes small eyes, accompanied by a decrease of retinal photoreceptor cells. Two genes encoding enzymes involved in RA synthesis are dramatically reduced in PBZ-treated eyes, indicating that PBZ impairs RA production. In addition, PBZ treatment reduces mitotic cells in the eye but does induce cell death. Furthermore, supplementation with RA partially rescued the reduced mitotic cells and eye size in PBZ-treated embryos. Our results revealed that PBZ exposure disrupts the retinoic acid signaling homeostasis in zebrafish embryos, leading to reduced proliferation of retinal photoreceptor cells. Furthermore, the PBZ-reduced eye size cannot be completely rescued by ectopic addition of RA, which suggests that not only retinoic acid signaling but also other signaling pathways may be involved in the toxic effects of PBZ on the eye. Future transcriptomic studies investigating the alteration of the expression of factors regulating retinal development will address this possibility. We expect that such future studies will help us better understand the potential toxicity of PBZ.
